# Essential oil of *Siparuna guianensis* as an alternative tool for improved lepidopteran control and resistance management practices

**DOI:** 10.1038/s41598-018-25721-0

**Published:** 2018-05-08

**Authors:** Adriano M. Lourenço, Khalid Haddi, Bergman M. Ribeiro, Roberto F. T. Corrêia, Hudson V. V. Tomé, Oscar Santos-Amaya, Eliseu J. G. Pereira, Raul N. C. Guedes, Gil R. Santos, Eugênio E. Oliveira, Raimundo W. S. Aguiar

**Affiliations:** 1 Departamento de Biotecnologia, Universidade Federal de Tocantins, Gurupi, TO 77413-070 Brazil; 2 Departamento de Entomologia, Universidade Federal de Viçosa, Viçosa, MG 36570-900 Brazil; 3 Departamento de Biologia Celular, Universidade de Brasília, Brasilia, DF 70910-900 Brazil; 4 EAG Laboratories, 13709 Progress Blvd #24 Suite S163, Alachua-FL, 32615 USA

## Abstract

Although the cultivation of transgenic plants expressing toxins of *Bacillus thuringiensis* (*Bt*) represents a successful pest management strategy, the rapid evolution of resistance to *Bt* plants in several lepidopteran pests has threatened the sustainability of this practice. By exhibiting a favorable safety profile and allowing integration with pest management initiatives, plant essential oils have become relevant pest control alternatives. Here, we assessed the potential of essential oils extracted from a Neotropical plant, *Siparuna guianensis* Aublet, for improving the control and resistance management of key lepidopteran pests (i.e., *Spodoptera frugiperda* and *Anticarsia gemmatalis*). The essential oil exhibited high toxicity against both lepidopteran pest species (including an *S*. *frugiperda* strain resistant to Cry1A.105 and Cry2Ab *Bt* toxins). This high insecticidal activity was associated with necrotic and apoptotic effects revealed by *in vitro* assays with lepidopteran (but not human) cell lines. Furthermore, deficits in reproduction (e.g., egg-laying deterrence and decreased egg viability), larval development (e.g., feeding inhibition) and locomotion (e.g., individual and grouped larvae walking activities) were recorded for lepidopterans sublethally exposed to the essential oil. Thus, by similarly and efficiently controlling lepidopteran strains susceptible and resistant to *Bt* toxins, the *S*. *guianensis* essential oil represents a promising management tool against key lepidopteran pests.

## Introduction

The worldwide adoption of genetically modified crop plants expressing toxic proteins of the bacterium *Bacillus thuringiensis* (i.e., *Bt* toxins) was a response to the economic losses caused by lepidopteran pest species, particularly in maize and soybeans^1^. However, despite the importance of genetically modified *Bt* plants on the integrated management of lepidopteran and coleopteran pests^2–4^, the sustainability of this agricultural practice has been threatened by lack of information about the interaction of *Bt* toxins (e.g., Cry proteins) with non-target organisms (including other relevant pests such as the phytosuccivorous insect pests) and the rapid evolution of high levels of resistance. Indeed, recent studies described field-evolved *Bt*-resistance in the fall armyworm *Spodoptera frugiperda* to non-pyramided *Bt* plants expressing Cry1F, Cry1A. 105, Cry2Ab and Cry1Ab toxins as well as to the dual-gene *Bt* (i.e., expressing Cry1A.105 and Cry2Ab toxins) maize^5–10^. Additionally, cases of cross-resistance between *Bt* toxins and non-*Bt* conventional insecticides were reported in the diamondback moth *Plutella xylostella* (i.e., pyrethroids and Cry1Ac toxin) and in *S*. *frugiperda* (i.e., organophosphates and Cry1F toxin)^11–14^.

Plant-derived products such as essential oils are regarded as a complementary alternative for the integrated pest management of insect pests, as long as they are effective and pose lower health and environmental risks than synthetic insecticides^15–18^. However, major threats to these plant-derived insecticides (or bioinsecticides) becoming commercially available are the lack of regulatory priorities and policies favoring their sustainable uses^16,17^, which seem to be a changing pattern scenario^19^.

The Neotropical region exhibits a diverse flora that remains largely underexploited as a source of biologically active substances. The aromatic and medicinal Neotropical plant species *Siparuna guianensis* Aubl. (Siparunaceae), also commonly referred to as Negramina, ant bush or Capitiú, is a relevant example. This plant species is widespread in South America, including the Brazilian Northeast and Mid-Western regions^20^, and products derived from its leaves, bark, and flowers have been used in folk medicine^21–23^. However, there are few investigations on the insecticidal activity of the essential oils and their constituents from the leaf, stem, and fruits of *S*. *guianensis*. Only recently, the essential oil of this plant species was explored for pest management with promising results against the mosquitos *Aedes aegypti* and *Culex quinquefasciatus*^24^, ticks^25^, and the wax moths *Achroia grisella* and *Galleria mellonella*^26^.

Thus, the present study aimed at shedding further light on the insecticidal activity of the *S*. *guianensis* essential oil. The chemical constituents of essential oils of *S*. *guianensis* were initially identified and subsequently tested on the velvetbean caterpillar *Anticarsia gemmatalis* (Hübner), a key soybean pest species, and on *Bt*-susceptible and resistant strains of the fall armyworm *Spodoptera frugiperda* (J.E. Smith), a key pest species of maize and soybeans. The larvicidal, ovicidal, egg-laying deterrence and repellence properties of the *S*. *guianensis* essential oil were assessed, as was the potential impairment of larval walking activity of both caterpillar species. We also assessed the toxicity of this essential oil on lepidopteran and human cell lines.

## Material and Methods

### Plant material, essential oil extraction and characterization

*Siparuna guianensis* was collected in the counties of Gurupi (11°43′45″ latitude S. 49°04′07″ longitude W) and Formoso do Araguaia (11°47′48″ latitude S. 49°31′44″ longitude W), State of Tocantins, Central Brazil. The collections were approved by the Brazilian National Council of Scientific and Technological Development (CNPq. n° 010580/2013–1). Taxonomic identification was carried out and confirmed by experts at the herbarium of the Federal University of Tocantins (Porto Nacional, TO, Brazil), where the samples were deposited under the reference number 10.496. The leaves of *S*. *guianensis* were collected in the mornings and used to extract the essential oils by hydrodistillation in a Clevenger apparatus as detailed elsewhere^24^.

The GC-MS analysis was performed on a Shimadzu QP-2010 instrument (Kyoto, Japan) operating at 70 eV with a DB-5MS methylpolysiloxane column (30 m × 0.25 mm × 1.0 μm; J & W Scientific Inc. Folsom. USA). The injection split ratio was 1:50 throughout the run (60.3 min) and helium was used as carrier gas at a flow rate of 1.50 mL/min (53.5 Kpa). The constant linear velocity was established at 42 cm/s and the injector temperature at 250 °C. The temperature of the transfer line was 260 °C.

The GC-FID analysis was performed on a Shimadzu GC-2010 Plus instrument (Kyoto, Japan), with a flame ionization detector (FID), and a CP-Sil column 8 CB with methylpolysiloxane as the stationary phase (30 m × 0.25 mm × 0. 25 μm (Varian Inc., Palo Alto, USA). The injection split ratio was 1:50 flow division throughout the run (60.3 min), and nitrogen was used as carrier gas with constant flow of 1.5 mL/min, an injector temperature of 250 °C, and a detector temperature of 260 °C.

The GC column oven temperature went from 70 °C to 180 °C at a rate of 4 °C/min, with a hold time of 27.5 min followed by a heating ramp of 25 °C/min to 250 °C, and a final hold time of 30 min^27^. The constituents of the oil were identified using standard reference compounds and by matching the mass spectra fragmentation pattern with the National Institute of Standards and Technology (NIST) Mass Spectra Library stored in the GC-MS database.

### Insects

Two populations of the fall armyworm *Spodoptera frugiperda* (*Bt* resistant and susceptible) and one of the velvetbean caterpillar *Anticarsia gemmatalis* (Lepidoptera: Noctuidae) were used in this study. The population of the fall armyworm resistant to the *Bt* toxins Cry1A.105 and Cry2Ab and a susceptible population of the velvetbean caterpillar were provided by the Insect-Plant Interaction Laboratory of the Federal University of Viçosa (Viçosa, MG, Brazil). The susceptible population of the fall armyworm was provided by the Laboratory of Integrated Pest Management of the Federal University of Tocantins (Gurupi, TO, Brazil).

### Concentration-mortality bioassays

Concentration-mortality bioassays were carried out using 3^rd^ instar larvae in a completely randomized experimental design. We used impregnated filter paper (9 cm in diameter) as the surface for the essential oil (contact) exposure. The essential oil of *S*. *guianensis* was dissolved in a mixture of water and 2% (v/v) of the detergent dimethyl sulfoxide (DMSO) to obtain the desired concentrations. Filter paper disks were impregnated with 300 µL of this solution and placed covering the inner walls of a 100 mL plastic cup, which received 25 larvae of the velvetbean caterpillar or a single larva of the armyworm (to avoid cannibalism). Each bioassay was replicated four times, and each replicate contained 25 velvetbean caterpillars or 16 armyworms. Larval mortality was recorded after 24 h of exposure, considering dead the larvae that were unable to walk when prodded with a fine hair brush. A similar procedure was used to establish the concentration-response curves for the synthetic insecticides indoxacarb (Rumo 300 g a.i./L; DuPont do Brasil S.A., Barueri, SP, Brazil) against the 3^rd^ instar larvae of all strains. The conventional insecticide was used as positive control for the assessed insecticidal activity of the essential oil of *S*. *guianensis*.

### Cultured cell viability

To analyze the effect of the essential oil of *S*. *guianensis* on the viability of lepidopteran cells, cultured cells from *S*. *frugiperda* [IPLB-SF-21AE;^28^] and from *A*. *gemmatalis* [UFL-AG-286;^29^] supplemented with 10% bovine fetal serum (Gibco-BRL) were maintained at 27 °C in TC-100 medium (Vitrocell; Campinas, SP, Brazil). In 96-well microplates, 10^4^ cells/well were incubated for a 24 h period with serial dilutions of *S*. *guianensis* essential oil at the concentrations of 0, 0.4, 0.04, 0.004, and 0.0004 µL**/**mL. Negative controls without the addition of the essential oil were also incubated for each cell line. All assays were carried out in triplicates. Cell viability was determined by the trypan blue exclusion method in the Countess Automated Cell Counter (Invitrogen; Carlsbad, CA, USA), using the manufacturer specified protocol. The same experiment was performed with a human monocytic cell line (TPH1) after incubation with increasing concentrations of the *S*. *guianensis* essential oil (0.85; 1.30; 1.70 and 2.12 µL/mL).

Finally, to investigate the potential cytopathic effects of *S*. *guianensis* essential oil on cultured lepidopteran cells, IPLB-SF-21AE and UFL-AG-286 cells were incubated with the essential oil at a concentration of 0.86 mg/mL. The culture medium was removed after the incubation period (i.e., 24 h) and the cells were immediately treated with reagents provided in the Apoptosis/Necrosis Detection Kit (blue, green, red) (Abcam®; Cambridge, UK), following the manufacturer’s instructions. The assayed cells were analyzed using a fluorescence microscope (Axiovert 100; Zeiss GmBh, Oberkochen, Germany).

### Ovicidal activity

The ovicidal activity assay was carried out according to the methods described in^30,31^, with the following modifications. The effect of the *S*. *guianensis* essential oil on the egg viability of *A*. *gemmatalis* and *S*. *frugiperda* was evaluated by immersing groups of 10 eggs for 30 seconds into a solution of the oil mixed with DMSO at 2% (v/v) in distilled water. The oil concentration used was equivalent to LC_10_ (i.e., *S*. *frugiperda*: LC_10_ = 3.34 μL/mL and *A*. *gemmatalis*: LC_10_ = 0.32 μL/mL). DMSO at 2% (v/v) in distilled water served as the control. A completely randomized experimental design was used with four replicates for *S*. *frugiperda* and ten replicates for *A*. *gemmatalis*. Egg viability (%) was recorded by counting the larva emergence after 72 h of exposure.

### Deterrence bioassay

The oviposition deterrence sparked by the *S*. *guianensis* essential oil was analyzed following previously described method^32^ with some modifications. PVC oviposition containers of 20 cm (diameter) per 25 cm (height), internally covered with sulfite paper, were used. Half of the internal area was covered by sulfite paper treated with 20 mL of the essential oil at a concentration equivalent to LC_10_ and allowed to air dry. The other half was treated with either DMSO 2% in distilled water or water only (as a control). Fifty containers were used per treatment and species (*S*. *frugiperda* and *A*. *gemmatalis*). Each container received a couple of adult moths (i.e., 1 ♀ and 1 ♂), and the number of eggs laid in each area was recorded after 48 h.

### Feeding inhibition bioassays

For the feeding inhibition effects of the *S*. *guianensis* essential oil, free- and no-choice bioassays were carried out using a leaf dip based protocol as described by^32^ with some adjustments. Plants of maize (variety AG1051 Agroceres, Santa Cruz das Palmeiras, SP, Brazil) and soybean (MSOY 8866, Monsanto, St. Louis, Missouri, USA) were grown in the greenhouse at the Horticulture Section of the Federal University of Tocantins (Gurupi, TO, Brazil). The maize and soybean leaves were obtained from 30-day-old plants. At the beginning of the experiment, 4 cm^2^ leaf sections as well as 3^rd^ instar larvae of *S*. *frugiperda* and *A*. *gemmatalis* were weighted separately. Each leaf section was dipped for 10 seconds in a 15 mL solution of the essential oil and then left to air dry at room temperature before being offered to individual larvae. The leaf sections and larvae were put in 30 mL plastic containers. In the free-choice bioassay, both treated and untreated leaf sections were offered simultaneously to the larvae. The two leaf sections were put on the bottom of the cup without touching each other.

In the no-choice bioassay, either a treated or an untreated leaf section was offered to a single larva in a single plastic container. The containers were kept closed with a plastic lid where small holes were made to allow gas exchange. After 24 h of exposure, the changes in the leaf sections and the larvae weights were recorded. The oil concentration equivalent to LC_10_ was used and the control treatment consisted of water and DMSO. The experimental design was completely randomized with 25 replicates per concentration; the experiment was replicated four times.

### Behavioral (locomotory) bioassays

The locomotory bioassays followed previously described methodologies with slight modifications^33–35^. Briefly, the larvae locomotory bioassays of both species (*S*. *frugiperda* and *A*. *gemmatalis*) were performed 3, 6, and 16 h after contact exposure to the respective LC_10_ and LC_50_ values of the *S*. *guianensis* essential oil determined for each Lepidoptera pest species (*S*. *frugiperda*: 3.34 (LC_10_) and 8.09 (LC_50_) μL/mL; *A*. *gemmatalis*: 0.32 (LC_10_) and 2.45 (LC_50_) μL/mL). The contact exposure was performed using filter paper impregnated with 1 mL of the oil solution (or control) and left to dry at air temperature. The exposed larvae were placed (either individually or in group) in an arena consisting of an uncontaminated filter paper placed in a Petri dish (135 × 20 mm); the movement of the larvae within the arena was recorded for 10 min using an automated video tracking system equipped with a CCD camera (ViewPoint Life Sciences, Montreal, Canada).

#### Individual locomotory bioassays

Two behavioral bioassays were carried out in arenas that were either fully treated or half-treated with essential oils. The third instar larvae of each studied population were exposed by contact to filter paper impregnated with the *S*. *guianensis* essential oil at a concentration equivalent to the LC_50_ for 3 h and subsequently submitted to individual walking bioassays. The 3^rd^ instar was chosen due to its greater walking activity compared to the other instars. No mortality was observed during the 3 h oil exposure period. In the bioassays with fully treated arenas, the procedure was similar to the one described for the group locomotory bioassay except that the walking activity was recorded for 30 min. The parameters recorded included the walked distance (cm), velocity (cm/s), resting time (s) and number of stops in the arena. Twenty larvae were used for each population. In each trial or replicate, the Petri dish and the filter paper were replaced. In the bioassays with half-treated arenas, the amount of time spent on the oil-treated and on the untreated zones were recorded and used to calculate the proportions of time spent on each side of the arena. Forty larvae were used for each population and essential oil treatment. In each trial or replicate, the Petri dish and the filter paper were replaced.

#### Group activity bioassays

The group activity bioassays were performed for the treated and untreated larvae of the 2^nd^, 3^rd^, 4^th^ and 5^th^ instar of each population. Ten replicates (i.e., groups of 10 larvae each) were used for each combination of instar and concentration. The general activity of the group (including walking, interaction between larvae, and movements of the body parts) within the arena was recorded for 10 min.

### Statistical analysis

Concentration–mortality curves were estimated using probit analysis with the PROBIT procedure in the SAS statistical software package^36^. The differential (essential oil and indoxacarb) susceptibility between lepidopteran species was estimated for each compound based on the estimated LC_50_ (i.e., the lethal concentration capable of killing 50% of tested lepidopterans) for each compound and lepidopteran species, and the tolerance ratios (TR_50_) were estimated by dividing the LC_50_ value obtained for *A*. *gemmatalis* by the LC_50_ value obtained for *S*. *frugiperda* strains^37^. The 95% confidence limits of these toxicity rate estimates were considered to be significantly different (*P* < 0.05) if they did not include the value 1^37^.

The results of egg viability, oviposition, foliar consumption and larval weight were subjected to univariate analysis of variance (ANOVA), or Kruskal-Wallis one-way ANOVA on ranks when the assumptions of normality and homoscedasticity were not satisfied. The results of walking behavior were submitted to multivariate analysis of variance (MANOVA) to secure an overall error level of *P* < 0.05 with subsequent (univariate) analyses of variance of each trait, when appropriate (PROC GLM;^36^). The results of group activity were subjected to repeated measure analyses of variance to determine the effects of essential oil concentrations. Differences were recognized by Fisher’s exact test (PROC ANOVA;^36^).

### Ethical approval

All applicable international, national, and institutional guidelines for the care and use of animals were considered in the present investigation.

### Informed consent

All the authors of this manuscript accepted that the paper is submitted for publication in the *Scientific Reports* journal, and report that this paper has not been published or accepted for publication in another journal, and it is not under consideration at another journal.

### Data availability statement

All relevant data are within the manuscript text and figures.

### Third party rights

All the figures and tables presented in the manuscript are originally constructed by the authors of the present manuscript authors not requiring any written permission for their use.

## Results

### Essential oil composition

The two major components of *S*. *guianensis* essential oils were the monoterpene β-myrcene (69.3–79.7%) and the ketone 2-undecanone (8.37–10.8%) (Table 1), although, there was a wide range of other compounds in smaller amounts in all of the three samples evaluated (Table 1).

**Table 1:** Chemical composition of *S*. *guianensis* essentials oil samples extracted from plants from different localities of the Gurupi (i.e., Gurupi 1 and Gurupi 2) and Formoso do Araguaia counties (Tocantins State, Central Brazil).

*Components*	*Gurupi 1*		*Gurupi 2*		*Formoso do Araguaia*	
	KI^a^	%(CG/DIC)	KI^a^	% (CG/DIC)	KI^a^	% (CG/DIC)
Santolina triene	927	t^b^	926	t^b^	926	t^b^
*α*-pinene	939	0.33	939	0.38	939	0.51
Camphene	957	0.10	956	0.11	957	0.12
*β*-pinene	984	0.15	984	0.17	984	0.20
*β*-myrcene	990	69.30	990	79.71	990	75.80
D-limonene	1034	0.58	1034	0.67	1034	0.74
*β*-ocimene	1050	0.57	1049	0.64	1050	0.47
Terpinolene	1089	0.15	1089	0.16	1089	0.14
2-Undecanone	1293	8.37	1293	9.58	1293	10.81
*β*-elemene	1389	0.37	1388	0.36	1388	0.51
*β*-cariofilene	1418	0.39	1418	0.35	1418	0.13
Germacrene D	1480	0.64	1480	0.80	1480	1.04
Byciclo-germacrene	1493	1.76	1493	1.21	1493	1.59
Germacrene A	1505	0.50	1504	0.42	1505	0.21
*γ*-cadinene	1517	0.10	1516	0.09	1514	0.19
Germacrene B	1557	0.21	1556	0.11	1557	0.15
Spatulenol	1575	0.21	1574	0.25	1574	0.62
Epi-α-cadinol	1645	0.25	1644	0.18	1644	0.32
*α*-cadinol	1654	0.19	1653	0.12	1653	0.26
*α*-copaene	—	—	1375	0.13	1375	0.25
Total		84.17		95.44		94.06

^a^Calculated Kovats retention indexes.

^b^t – Traces quantity (<0.1%).

### Concentration-mortality bioassays

The estimated concentration-mortality parameters obtained using the probit model were appropriate based on the low χ^2^-values (<8.0) and high *P*-values (>0.05) (Table 2). The LC_50_ for *A*. *gemmatalis* was 2.45 µL of essential oil/mL, while for *S*. *frugiperda* it was 8.09 µL of essential oil/mL (for the Cry1A.105 and Cry2Ab susceptible strain) and 7.11 µL of essential oil/mL (for the Cry1A.105 and Cry2Ab resistant strain). Based on the LC_50_ measures obtained from these concentration–mortality bioassays, *S*. *frugiperda* was more tolerant (i.e., TR_50_ ranged from 2.5 to 4.0-fold) than *A*. *gemmatalis* (i.e., TR_50_ ranged from 2.5 to 4.0-fold) to the *S*. *guianensis* essential oil (Table 2).

**Table 2:** Relative susceptibility of key lepidopteran pests (i.e., *S*. *frugiperda* and *A*. *gemmatalis*) to the essential oil of *S*. *guianensis* and the synthetic insecticide indoxacarb (oxadiazine).

*Insecticide type*	*Lepidopteran strain*	*N*	Slope ± SEM	LC50 (95% FI) (µL/mL)	TR_50_* (95% CL)	χ^2^	*P*
Essential oil of *S*. *guianensis*	*S*. *frugiperda* (Cry1A.105 and Cry2Ab susceptible strain)	600	4.33 ± 0.61	8.09 (7.59–9.87)	3.3 (3.1–4.0)	0.49	0.97
	*S*. *frugiperda* (Cry1A.105 and Cry2Ab resistant strain)	600	1.67 ± 0.13	7.11 (6.94–7.28)	2.9 (2.5–3.1)	1.26	0.73
	*A*. *gemmatalis*	590	1.75 ± 0.18	2.45 (2.10–2.97)	1.0 (0.9–1.2)	2.90	0.57
*Indoxacarb* *(oxadiazine)*	*S*. *frugiperda* (Cry1A.105 and Cry2Ab susceptible strain)	570	1.93 ± 0.21	0.003 (0.002–0.006)	0.04 (0.02–0.08)	0.44	0.70
	*S*. *frugiperda* (Cry1A.105 and Cry2Ab resistant strain)	600	2.19 ± 0.21	0.005 (0.003–0.007)	0.06 (0.05–0.07)	6.2	0.82
	*A*. *gemmatalis*	540	1.60 ± 0.18	0.08 (0.06–0.10)	1.0 (0.8–1.1)	4.66	0.19

*Tolerance ratio estimated by dividing the LC_50_ value obtained for *S*. *frugiperda* strains by the LC_50_ value obtained for *A*. *gemmatalis* strain.

In contrast, there was an inverse susceptibility pattern of the insect species to the synthetic insecticide indoxacarb; its LC_50_ for *A*. *gemmatalis* was 0.08 µL a.i./mL, while for *S*. *frugiperda* it was 0.003 µL a.i./mL (for the Cry1A.105 and Cry2Ab susceptible strain) and 0.005 µL a.i./mL (for the Cry1A.105 and Cry2Ab resistant strain). Based on these LC_50_ estimates, *S*. *frugiperda* was less tolerant to indoxacarb than *A*. *gemmatalis* (i.e., TR_50_ ranged from 16.0 to 26.7-fold) (Table 2). Nonetheless, the essential oil toxicity was lower than that of indoxacarb (approximately 3.5-fold for *A*. *gemmatalis* and between 104.0 and to 379.5-fold for *S*. *frugiperda*).

### Ovicidal bioassays

**Figure 1:**
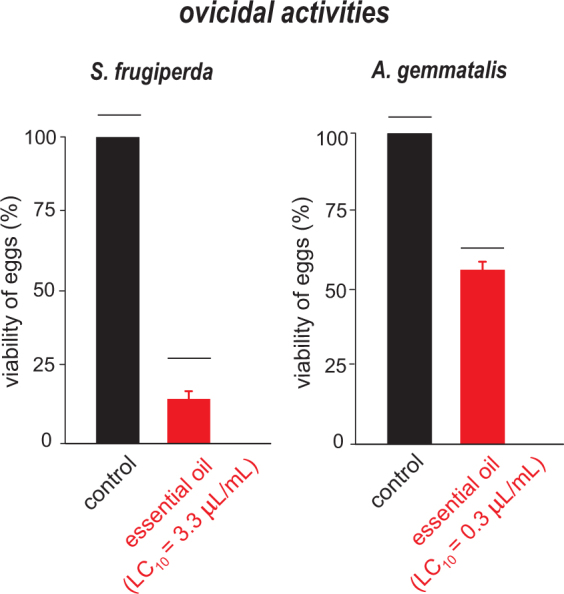
Viability of eggs of *Spodoptera frugiperda* and *Anticarsia gemmatalis* unexposed (control) and exposed to sublethal dose (LC_10_) of the essential oil of *Siparuna guianensis*. Horizontal bars indicate significant differences (*P* < 0.05) between exposed and unexposed eggs.

**Figure 2:**
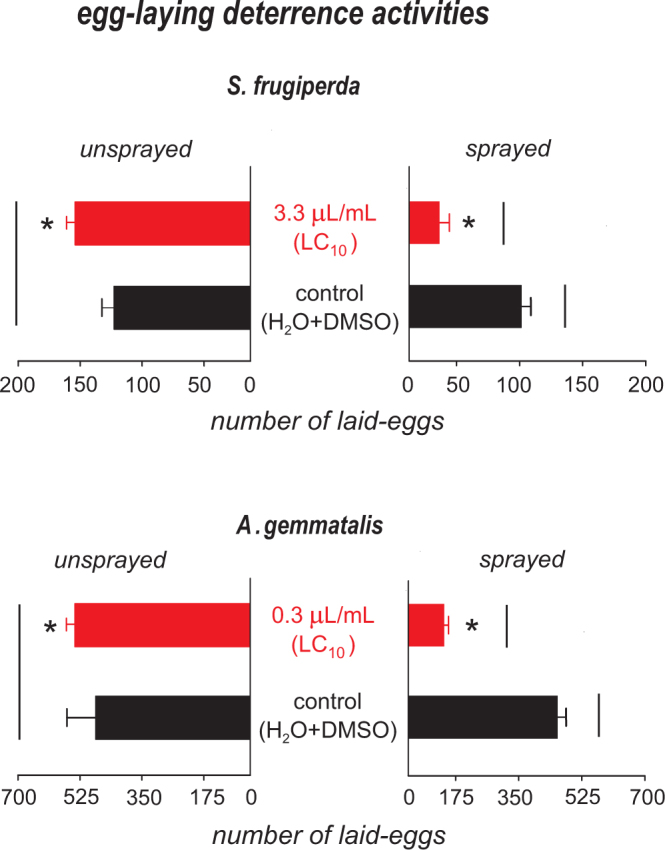
Number of eggs laid by females of *Spodoptera frugiperda* and *Anticarsia gemmatalis* on the sprayed with sublethal dose of the essential oil of *Siparuna guianensis* (LC_10_) and the unsprayed (H2O + DMSO) sides of the oviposition containers. Asterisks indicate significant differences (*P* < 0.05) between sprayed and unsprayed sides of the same treatment. Horizontal bars indicate significant differences (*P* < 0.05) between the same sides of the essential oil of Siparuna guianensis and control.

The *S*. *guianensis* essential oil significantly reduced egg viability of *A*. *gemmatalis* and *S*. *frugiperda* (Fig. 1). The effect on egg viability was higher for *S*. *frugiperda*, as the egg treatment resulted in less than 20% viability (Fig. 1A), while for *A*. *gemmatalis*, the egg viability was reduced by approximately 40% (Fig. 1B). The essential oil of *S*. *guianensis* also exhibited strong deterrence as adult female moths from both species preferred the untreated side of the container for egg-laying (*S*. *frugiperda: F*_*(1*,*48)*_ = 101.01; *P* < 0.001; *A*. *gemmatalis: F*_*(1*,*48)*_ = 34.10; *P* < 0.001) (Fig. 2). The number of eggs in the treated side was smaller than in the control by at least 80% for the concentration used (LC_10_).

### Cultured cell viability

**Figure 3:**
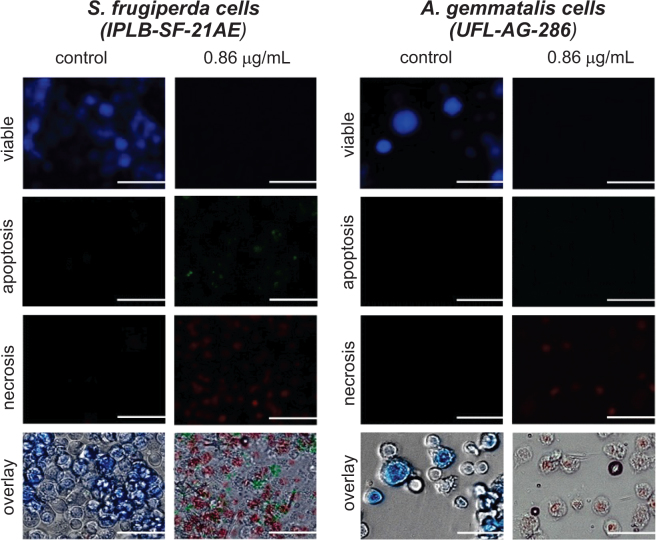
Cytopathic effects of the essential oil of *Siparuna guianensis* (0.86 mg/mL) on the viability of lepidopteran cultured cells from *Spodoptera frugiperda* (IPLB-SF-21AE) and *Anticarsia gemmatalis* (UFL-AG-286) visualized under fluorescence microscopy.

**Figure 4:**
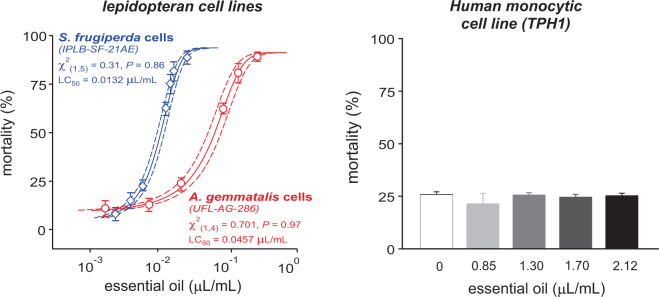
Toxicity of the essential oil of *Siparuna guianensis* to lepidopteran cells from *Spodoptera frugiperda* (IPLB-SF-21AE) and *Anticarsia gemmatalis* (UFL-AG-286) and to Human monocytic cell line (TPH1).

We tested the *in vitro* toxicity of the essential oil of *S*. *guianensis* on the viability of lepidopteran cultured cells from *S*. *frugiperda* (IPLB-SF-21AE) and *A*. *gemmatalis* (UFL-AG-286) incubated for a 24 h period with a concentration of 0.86 mg/mL of the essential oil. The cells from both species suffered severe alterations in their viability after the incubation period. The armyworm cells showed both necrotic and apoptotic death, while only necrosis seemed to be causing death of *A*. *gemmatalis* cells (Fig. 3). The *S*. *guianensis* essential oil exhibited higher toxicity against the *S*. *frugiperda* than *A*. *gemmatalis* cell lines (Fig. 4), but mortality and toxic effects were not observed in the human monocytic cell line (TPH1) incubated with increasing concentrations of the *S*. *guianensis* essential oil (Fig. 4). However, it is worth noting that the lowest tested concentration (i.e., 0.85 µL of essential oil/mL) was 85-fold higher than the LC_99_ estimated for the insect cultured cells (IPLB-SF-21AE and UFL-AG-286) (Fig. 4).

### Feeding inhibition bioassays

**Figure 5:**
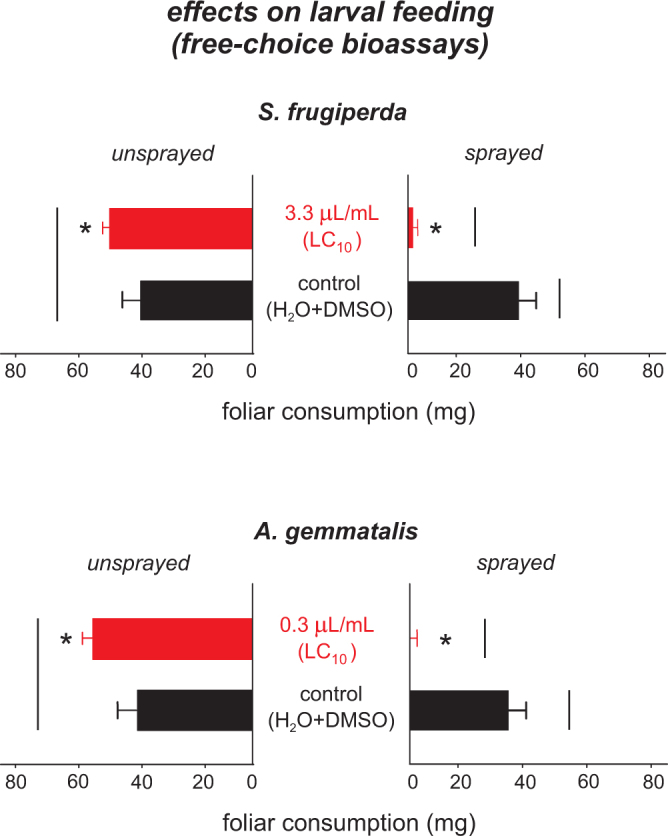
Foliar consumption (mg) of the 3^rd^ instar larvae of *Spodoptera frugiperda* and *Anticarsia gemmatalis* feeding on sprayed with sublethal dose (LC_10_ of *Siparuna guianensis* essential oil) and unsprayed leaves of maize or soybean. Asterisks indicate significant differences (*P* < 0.05) between sprayed and unsprayed leaves of the same treatment. Horizontal bars indicate significant differences (*P* < 0.05) between the leaves sprayed either with the essential oil of *Siparuna guianensis* or unsprayed (control).

**Figure 6:**
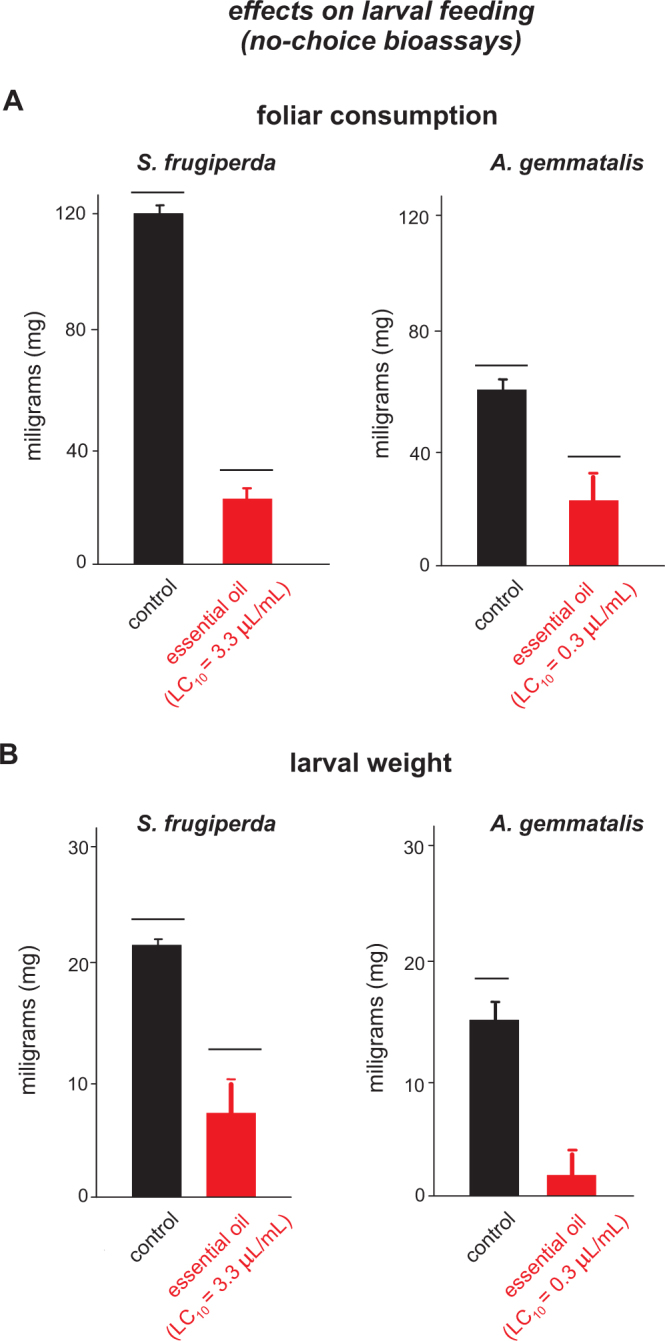
Foliar consumption (mg) and weight gain of the 3^rd^ instar larvae of *Spodoptera frugiperda* and *Anticarsia gemmatalis* feeding on maize or soybean leaves in no choice experimental design. Horizontal bars indicate significant differences (*P* < 0.05) between sprayed with sublethal dose (LC_10_) and unsprayed leaves maize and soybean.

In the free-choice feeding bioassays, the feeding activity of 3^rd^ instar *S*. *frugiperda* and *A*. *gemmatalis* larvae on the treated leaves was significantly lower than the untreated ones. Larvae completely avoided feeding on the leaves of maize and soybean treated with *S*. *guianensis* essential oil (Fig. 5). Furthermore, in the no-choice experiments, both species showed significantly reduced feeding activity on the leaf sections treated with essential oil of *S*. *guianensis* compared to the controls (Fig. 6A), which influenced negatively the weight gain of all larvae that were submitted to these treated sections (Fig. 6B).

### Behavioral (locomotory) bioassays

#### Individual locomotory bioassays

**Figure 7:**
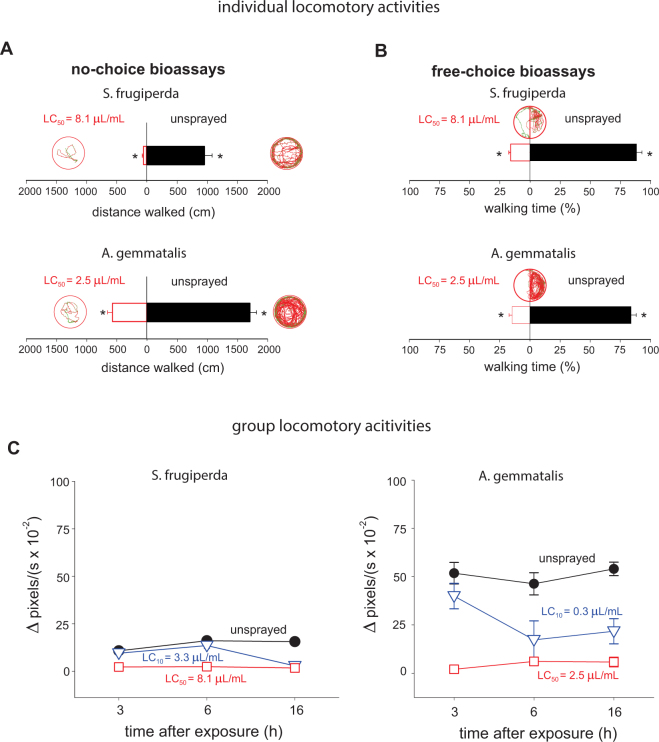
Individual walking behavior (**A**,**B**) and group activity (**C**) of 3^rd^ instar larvae of *Spodoptera frugiperda* and *Anticarsia gemmatalis* exposed to *Siparuna guianensis* essential oil in arenas fully treated and untreated (**A**,**C**) or half-treated (**B**). Asterisks indicate significant differences (*P* < 0.05) between exposed and unexposed arenas.

The multivariate analysis of variance showed that the walking behavior of the 3^rd^ instar larvae was significantly influenced by the essential oil of *S*. *guianensis* (Table 3). This alteration in walking behavior was best seen in the distance walked as the larvae of all the populations tended to walk shorter distances when in contact with treated surfaces (Fig. 7A). In the free choice bioassays, the larvae of the two lepidopteran pests spent significantly more time in the untreated area (more than 80%) compared to the treated area indicating a strong repellence to the essential oil (*S*. *frugiperda*: *t* = 17.05; *df* = 39; *P* < 0.001; *A*. *gemmatalis: t* = 15.09; *df* = 39; *P* < 0.001) (Fig. 7B).

**Table 3:** Results of the analysis of variance for the general activity of 3^rd^ instar larvae groups of key lepidopteran pests (i.e., *S*. *frugiperda* and *A*. *gemmatalis*) after 3, 6 and 16 h of exposure to the LC_10_ or LC_50_ of the *S*. *guianensis* essential oil estimated for each lepidopteran pest.

Variation source	*df*	*Distance walked*		*Stopping time*		*Velocity*		*Number of stops*	
		F	*P*	F	*P*	F	*P*	F	*P*
Species (S)	2	11.77	<0.001*	45.71	<0.001*	1.28	0.28	41.90	<0.001*
Essential oil Concentration (EOC)	1	117.71	<0.001*	266.04	<0.001*	0.01	0.93	17.72	<0.001*
S X EOC	2	3.22	0.04*	5.89	0.003*	8.49	0.0003	5.87	0.003*

*S*: insect species (i.e., *S*. *frugiperda* and *A*. *gemmatalis*); EOC: LC_10_ and LC_50_ values of the *S*. *guianensis* essential oil estimated for each lepidopteran pest.

#### Group activity bioassays

The activity bioassays demonstrated that the general activity of the larvae groups was significantly (Table 4) influenced by the exposure to the essential oil (Fig. 7C). Changes in group behavior over time were found for all tested populations when exposed to either LC_10_ or LC_50_ compared to the unexposed control. The 3 h exposure to LC_50_ of the essential oil already resulted in a drastic reduction of the group activity. This reduction was persistent after 6 and 16 h. Similar trends were found for the sublethal dose of LC_10_, especially after 16 h of exposure.

**Table 4:** Results of the multivariate analysis of variance for the walking behavior of the 3^rd^ instar larvae of key lepidopteran pests (i.e., *S*. *frugiperda* and *A*. *gemmatalis*) in arenas treated with either the LC_10_ or LC_50_ of the oil of *S*. *guianensis*.

Sources of variation	df	Grouped larval locomotory activities		
		F	*P*	
**Between samples**				
Species (S)	1	66.4	<0.0001*	
Essential oil concentration (EOC)	2	69.8	<0.0001*	
S x EOC	2	23.3	<0.0001*	
Error	74	—	—	
	**df**_**den**_ **/df**_**num**_	**Wilks’ lambda**	**F**_**approx**_	***P***
**Within samples**				
Time (T)	3/72	0.80	13.28	<0.001*
T x S	3/72	0.74	8.64	<0.001*
T x EOC	4/146	0.81	5.82	<0.001*
T x S x EOC	4/146	0.80	3.09	0.002*

*T*: time of exposure (i.e., 3, 6 and 16 h); *S*: insect species (i.e., *S*. *frugiperda* and *A*. *gemmatalis*); *EOC*: LC_10_ and LC_50_ values of the *S*. *guianensis* essential oil estimated for each lepidopteran pest.

## Discussion

Some plant extracts, especially essential oils, have been shown to exhibit insecticidal activities. They are potential alternative products for insect pest control since they are biodegradable and environmentally safer than several conventional insecticides^16–18,38,39^. Here, we report on the high toxicity of the essential oil of a Neotropical plant, the Negramina *S*. *guianensis*, to the larval stages of key lepidopteran pests (i.e., *A*. *gemmatalis* and *S*. *frugiperda*, including a *S*. *frugiperda* strain that is resistant to the Cry1A.105 and Cry2Ab *Bt* toxins). This high toxicity was associated with induction of necrotic and apoptotic cell death in lepidopteran cells revealed by *in vitro* assays, which were absent from the human monocytic cell line TPH1. We also report that the sublethal exposure to *S*. *guianensis* resulted in deficits in reproduction (e.g., egg-laying deterrence and decreased egg viability), larval development (e.g., feeding inhibition) and locomotion (e.g., individual and grouped larvae walking activities).

Our chromatographical analyses revealed the monoterpene β-myrcene (varying from approximately 69% to 80%) and the non-terpenic acyclic ketone 2-undecanone (varying from approximately 8% to 11%) as the major components of the *S*. *guianensis* essential oil obtained from different samples. Although these results are similar to those from previous studies^21,24,26^, they also differed from chemical profiles reported for *S*. *guianensis* essential oils extracted from plants collected from other Brazilian regions^24,25,40–43^. These findings confirm the spatio-temporal variations (e.g., temperature, relative humidity, photoperiod, irradiance, genotype, extraction method and agronomic conditions) commonly encountered in the chemical composition of essential oils and that can influence the chemical profile of essential oil extracted from the same plant species^18,44–46^.

In the present investigation, the *S*. *guianensis* essential oil exhibited insecticidal toxicity towards the *A*. *gemmatalis* and the *S*. *frugiperda* (including a strain that is resistant to Cry1A.105 and Cry2Ab *Bt* toxins). Such activity is suggestive of their potential as insect pest control agents, although their potency is lower than that of the oxadiazine indoxacarb. However, the essential oil activity may well be enhanced with the use of adjuvants and with oral exposure in addition to contact, as demonstrated in our feeding bioassays. The activity of *S*. *guianensis* essential oil was also previously reported for other pest species including the wax moths *G*. *mellonella* and *A*. *grisella*^26^, the mosquitoes *A*. *aegypti* and *C*. *quinquefasciatus*^24^ and the cattle tick *R*. *microplus*^25^. These previous investigations have attributed the *S*. *guianensis* essential oil toxicity to the actions of the oil’s major components (i.e., β-myrcene and 2-undecanone). Indeed, both β-myrcene^24,47–49^ and 2-undecanone^50–54^ have been previously reported to produce toxic effects across several insect and mite species. However, as some previous investigations suggest that substantial differences on the biological activities of the essential oils derived from subtle differences in oil components^18,46^, the precise contribution of each *S*. *guianensis* essential oil component for the insecticidal activities reported here still needs further toxicological investigation.

Although the mode of action of the *S*. *guianensis* essential oil was not addressed in detail here, and as previously described for the C6/36 mosquito cell lines^24^, our *in vitro* bioassays with this essential oil induced necrotic and apoptotic cell death on lepidopteran cultured cells. These effects might be derived from the actions of terpenes (e.g., the monoterpene β-myrcene) present in the essential oil. Terpenes generally allow the essential oil to diffuse through the cell membrane, causing not only cell membrane alterations but also uncoupling oxidative phosphorylation and electron transfer inhibition^55,56^. This interference on the composition or tridimensional arrangement of the plasma membrane can alter the basic structure for homeostatic balance and optimal physiological functioning^55^, and such damages may ultimately lead to cell death^57^. Intriguingly, our results showed that the viability of a human monocytic cell line (TPH1) was not altered by the *S*. *guianensi*s essential oil, which suggests the existence of differential target susceptibilities between vertebrate and insects cells. A recent investigation^26^ reported that the *S*. *guianensi*s essential oil selectively acts on two lepidopteran moth pests (i.e., *G*. *mellonella* and *A*. *grisella*) without affecting honey bees *Apis mellifera*, reinforcing the hypothesis of existing significant differences in the *S*. *guianensi*s essential oil target susceptibility, even between insect species.

Indeed, essential oils with high monoterpene contents have been shown to exert their action on octopamine, tyramine, GABA and TRP channels^58–62^. TRP channels are of particular interest as these transmembrane proteins enable individual cells to sense changes in their internal (i.e., osmolarity and fluid flow) and external environment^63,64^. It has been demonstrated that the TRPM-dependent Ca^2+^ influx may account for necrotic and apoptotic death in many cell types following changes in the redox state^63^, which would help to explain the cytopathic effects described here for the lepidopteran cells exposed to the *S*. *guianensi*s essential oil. Furthermore, as the TRP channels play a key role in calcium entry in excitable cells (e.g., muscles and neurons), altering the cytosolic free Ca^2+^ concentration and, consequently, the subcellular processes dependent of free Ca^2+^ concentration (e.g., muscle contraction and locomotion)^64,65^, it is reasonable to suggest that the lepidopteran TRP channels were indeed affected by *S*. *guianensis* essential oil.

Essential oil of *S*. *guianensi*s also induced oviposition deterrence and repellence, high feeding inhibition and reduced locomotion in populations of both *S*. *frugiperda* and *A*. *gemmatalis*, indicating strong alterations in the sensory mechanisms related to insect taste, smell and locomotion. These sensory traits are critical for multiple animal behaviors such as discriminating safe from noxious foods, detecting toxic odors and selecting sites for egg-laying^66^. The repellence of essential oils are largely linked with monoterpenes and phenolic compounds^38,67^. For instance, repellent actions of *S*. *guianensi*s essential oils have been reported against adult mosquitoes^24^ and larvae and adult lepidopteran pests^26^. Furthermore, sublethal exposure to monoterpene-rich essential oils reduced feeding and oviposition on *A*. *gemmatalis*^32^ and caused relevant alteration in important behaviors in other insect species (e.g., deterrent effects on larval feeding and adult oviposition in *Plutella xylostella*^68^ and altered walking activities in the maize weevils *Sitophilus zeamais*)^34,69^.

Thus, the findings described here not only revealed the potential control of two key lepidopteran pests (*S*. *frugiperda* and *A*. *gemmatalis*, including a *S*. *frugiperda* that is resistant to Cry1A.105 and Cry2Ab *Bt* toxins) by the *S*. *guianensi*s essential oil but also showed that this oil induced cell death (i.e., apoptotic and necrotic cells) in insect cells, although not in human cells. Such characteristics demonstrated that the current pest management initiatives will likely benefit from including *S*. *guianensi*s essential oil as an alternative tool in managing lepidopteran pests, especially in the context of raising concerns regarding the sustainable use and efficacy of *Bt*-plants. Further investigations aiming to elucidate the molecular interactions between the *S*. *guianensi*s essential oil (and its constituents) and the potential ionic channels (e.g., octopamine, tyramine, GABA and TRP) targeted by these molecules will shed light on the major mode of actions of these plant products on lepidopteran pests.
